# Identification and Validation of an Apoptosis-Related Gene Prognostic Signature for Oral Squamous Cell Carcinoma

**DOI:** 10.3389/fonc.2022.889049

**Published:** 2022-06-13

**Authors:** Shuqin Wang, Sien Zhang, Zhi Lin, Jingxin Ma, Lijun Zhu, Guiqing Liao

**Affiliations:** ^1^ Hospital of Stomatology, Guanghua School of Stomatology, Sun Yat-sen University, Guangdong Provincial Key Laboratory of Stomatology, Guangzhou, China; ^2^ Department of Oral and Maxillofacial Surgery, Guangdong Provincial People’s Hospital & Guangdong Academy of Medical Sciences, Guangzhou, China; ^3^ Department of Stomatology, The Sixth Affiliated Hospital, Sun Yat-sen University, Guangzhou, China; ^4^ School of Stomatology, Southern Medical University, Guangzhou, China

**Keywords:** oral squamous cell carcinoma, apoptosis-related genes, prognosis, nomogram, the cancer genome atlas

## Abstract

To identify an apoptosis-related gene (ARG) prediction model for oral squamous cell carcinoma (OSCC), we analyzed and validated the data from TCGA and GEO, respectively. Kaplan–Meier survival analysis and ROC curves showed a good prognostic ability of the model both in the internal training set and in the external testing set. Furthermore, we built a nomogram using these ARGs to forecast the survival probability of OSCC patients. Moreover, we evaluated the rate of immune cells infiltrating in the tumor samples and found obvious, different patterns between the high and low risk groups. GO and KEGG analyses demonstrated multiple molecular biological processes and signaling pathways connecting with this prognostic model in OSCC. The expression of these risk genes in clinical specimens was higher in the non-survival patients than in the well-survival patients by immunohistochemical staining analysis. In conclusion, we established a signature made up of six risk apoptosis-related genes to predict the survival rate of OSCC. These genes could also be targets for the treatment of OSCC.

## Introduction

In 2020, almost 377,713 new cases and 177,757 deaths of OSCC occurred all around the world (1). Traditionally, risk factors for OSCC include tobacco smoking, chewing betel nut, alcohol, excessive sunlight exposure, HPV infection, and poor oral hygiene (2).The primary treatment for oral squamous cell cancer (OSCC) patients is surgical resection plus chemotherapy and radiotherapy. Despite of the advancements in all these treatments nowadays, the 5-year survival rate remains poor (3). For decades, attempts to improve the prognosis have failed because of the complicated heterogeneity of tumors. Several patients are still over- or undertreated due to the unsatisfactory forecasting efficiency of conventional prognostic indicators and the uniformity of therapy guidelines (4). Currently, no molecular subtype that can guide individual targeted therapy has been recognized. Therefore, researchers are eager to identify the specific carcinogenesis and prognostic genes of OSCC.

Apoptosis or programmed cell death (PCD) is an evolutionarily conserved process to promote the development of organisms and keep the balance of tissue homeostasis (5). This progress is described as the cell suicide process. Overactivation or inactivation of apoptosis leads to diseases, such as Parkinson’s disease and tumorigenesis (6, 7). Escaping from apoptosis was considered as one of the 10 hallmarks of cancer. A great number of studies have indicated the key role of apoptosis in tumorigenesis and chemotherapy resistance (8). Researchers tried to restore each defect of the apoptosis signaling pathway and wiped out the cancer cells. They are focused on targeting the anti-apoptotic Bcl-2 family member (9), p53 (10), c-FLIP, and caspase families (11) (12). For example, myeloid cell leukemia-1 (MCL1), one of the anti-apoptotic proteins, has been identified as a prospective therapeutic target. Previous studies showed the potential value of apoptosis-related genes or proteins such as Bcl-2 and Survivin in the diagnosis and treatment of OSCC (13).

Our research hopes to identify the key prognostic genes and establish the core network of OSCC by using comprehensive bioinformatics analysis based on apoptosis-related genes.

## Materials and Methods

### The Collection of Apoptosis-Related Genes

We extracted the apoptosis-related gene sets from the Gene Set Enrichment Analysis (GSEA) dataset (http://www.gsea-msigdb.org/gsea/index.jsp), Reactome dataset (https://reactome.org/), and KEGG dataset (https://www.kegg.jp/kegg/). A total of 438 apoptosis-related genes (ARGs) were picked out and used for analysis.

### Data Acquisition

We got the RNA sequences and clinical information of 213 OSCC patients and 16 healthy human from TCGA database. Another dataset including 97 OSCC patients with complete follow-up data (GSE41613) was extracted from the GEO database. The data from TCGA were recognized as an internal training dataset, and the data from GEO were used as an external testing dataset.

### Identification of the Prognostic ARGs and Construction of the Prognostic Model

In TCGA dataset, we conducted univariate Cox analysis and Kaplan–Meier (K-M) survival analysis to seek ARGs associated with overall survival (OS) of OSCC. When ARGs met the criteria of p-value <0.05 in the two tests referred above, they were recognized as prognosis-related ARGs. These ARGs were selected for least absolute shrinkage and selection operator (LASSO). Next, we used multiple Cox analyses to select the independent ARGs. The risk score could be calculated by the following formula: Risk score=*Expression level of gene*1 × β1 + *Expression level of gene*2 × β2 + *Expression level of gene*3 × β3 + ⋯. 

β represents the coefficient. Therefore, we can acquire the risk score of OSCC patients easily. Based on the median risk score, all the patients were assigned into low-risk and high-risk groups. Then, K-M analysis was conducted to value the difference in survival rate between these two groups. In the study, the receiver operating characteristic (ROC) curve was applied. The value of the corresponding area under the ROC curve (AUC) could be used to test the sensitivity of this model and compare the forecasting accuracy with traditional clinical factors (14). In addition, we conducted univariate and multivariate analyses to assess the independent ability for predicting prognosis. Moreover, the external testing dataset from GEO was used to check the prognostic capability of the model.

### Construction of Nomogram

Furthermore, we created a nomogram to predict the OS of the OSCC patients on the basis of the independent prognostic ARGs. A calibration curve was applied to assess the efficiency of the nomogram. Finally, we verified the prognostic nomogram in the external testing dataset in the same way.

### Tumor-Infiltrating Immune Cell Analysis

To assess the infiltration pattern of immune cells in two risk groups, we applied the CIBERSORT analysis (15). In order to know more about the relationship between the immune microenvironment and apoptosis, we conducted the correlation analysis of nine types of immune cell and risk score.

### Functional Enrichment Analysis

We conducted Gene Ontology (GO) and Kyoto Encyclopedia of Genes and Genomes (KEGG) analyses for these six ARGs. These analyses discovered a series of molecular biological process and multiple signaling pathways *via* the R package clusterProfiler 4.0 (16). Moreover, an interaction network was built to display the correlation of six ARGs.

### Unsupervised Clustering Analysis

Unsupervised clustering analysis was used to distinguish patient subgroups with different apoptosis modification patterns based on the expression of the six ARGs. With the “Consensus Cluster Plus” R package, patients were assigned by k-means, with k from 2 to 9. On the grounds of the dispersion of the resulting consensus clustering matrix, a cumulative distribution function (CDF) curve, and the likelihood ratio, we obtained the optimal number of clusters. Moreover, we compared the different prognoses among the apoptosis clusters with K-M analysis.

### The Pan-Cancer Analysis

We acquired the expression data of the six genes in pan-cancer through GEPIA2. Then, we got the Cox proportional hazard ratio by R survival package to explore the relationship of the six genes and OS of pan-cancer.

### Immunohistochemical Staining and Evaluation

Immunohistochemical (IHC) staining of MCL1, GPI, and ARHGAP10 was performed using rabbit polyclonal anti-MCL1, anti-GPI, anti-ARHGAP10 antibodies (the concentration was 1:2,000, Cat. 16225-1-AP, 15171-1-AO, 55139-1-AP, respectively, Proteintech, Wuhan, China). All sections were scanned on an Aperio AT2 scanner (Leica Biosystems, Wetzlar, Germany) with a ×20 objective lens. We used Aperio ImageScope software to obtain the digital images of sample sections. Five random fields of the same size were selected in each slide. The H-score was carried out using the Aperio ImageScope software (17). All the samples were acquired from the first oral cancer radical surgery of the patients who had not received radiotherapy or chemotherapy yet. The patients who were diagnosed with oral squamous cell carcinoma for the first time were free of other cancers.

### Statistical Analysis

We conducted all statistical analyses and graphs through R software. Cox proportional hazard regression analysis was used for univariate and multivariate analyses. Overall survival times were calculated by the Kaplan–Meier analysis. The H-scores of different groups were compared with paired T test, and significant difference was considered when p < 0.05.

## Result

### Selection of Crucial Genes and Identification of ARGs Associated With OSCC Survival

**Figure 1:**
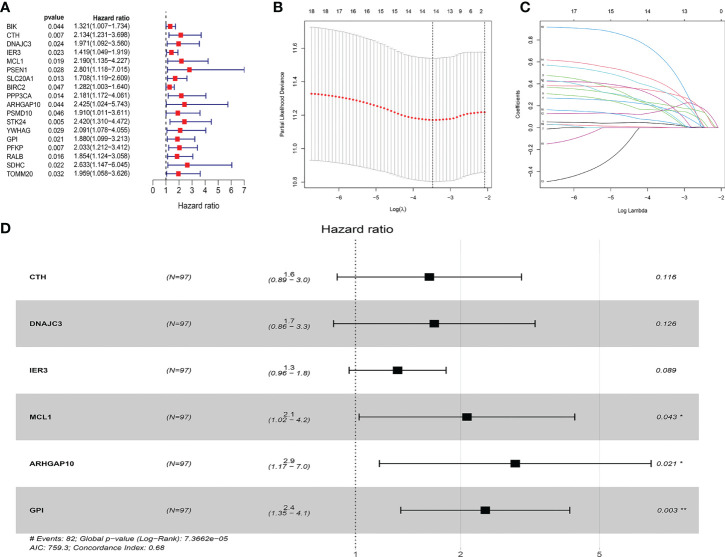
Construction of the ARG model in the internal training dataset. **(A)** Forest plot showed that 18 risk genes were identified as prognostic ARGs through univariate Cox regression analysis (HR >1, p < 0.05). **(B, C)** LASSO analysis further picked up 14 ARGs. **(D)** The multiple Cox regression screened six independent predictors (CTH, DNAJC3, IER3, MCL1, GPI, ARHGAP10) of OSCC.

We collected 438 ARGs from the GSEA gene set and the Reactome and KEGG databases. Based on the criteria set as p < 0.05, and hazard ratio >1, 18 ARGs were selected which are shown in the forest plot (**Figure 1A**). These hub genes were incorporated in LASSO analysis. Detailed information of the LASSO model optimal parameter and LASSO coefficient profiles is displayed in **Figures 1B** **, C**. Then, we used the multivariate Cox regression analysis to filter these candidates and constructed a prognostic signature. Finally, six ARGs (CTH, DNAJC3, IER3, MCL1, GPI, ARHGAP10) were identified to establish an apoptosis-related signature. Among these genes, MCL1, GPI, and ARHGAP10 are associated with a higher risk than others because of the higher hazard ratios (**Figure 1D**).

### Prognosis Model Construction and Risk Score Analysis

**Figure 2:**
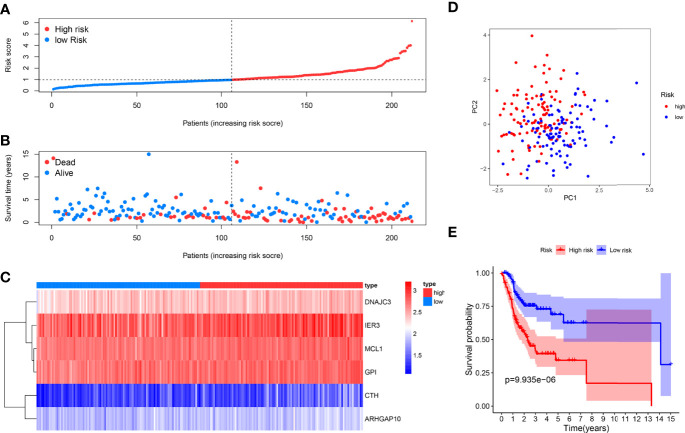
Prognosis analysis of the internal training dataset. **(A)** The median risk score allocated OSCC patients in TCGA into two groups. From left to right, the risk scores were ascending and each dot represents an individual. **(B)** Patients with different survival times and statuses were arranged with the increasing risk score from left to right; clearly, the dead patients have higher risk score. **(C)** The heatmap showed expression profile of the six risk genes. The expression level from high to low was manifested with the colors from red to blue. **(D)** The high- and low-risk groups were gathered in two directions through the PCA analysis. **(E)** Kaplan–Meier analysis showed different prognosis between the two groups.

We used the six prognostic genes selected above as independent factors to construct the prediction model. The expression value of six genes could define the risk score accurately: Risk score = (0.4874×expression value of CTH) + (0.5198×expression value of DNAJC3) + (0.2777× expression value of IER3) + (0.7352×expression value of MCL1) + (1.0540×expression value of ARHGAP10) + (0.8561×expression value of GPI). The median risk score allocated patients into low-risk and high-risk groups (**Figure 2A**). The results showed that the low-risk group has a longer survival time than the high-risk one (**Figure 2B**). A heat map reveals the different expression profile of six ARGs (**Figure 2C**). The patients in the low- and high-risk groups were gathered from different directions (**Figure 2D**). In addition, K-M analysis also supported that the low-risk group had a higher probability to survive (**Figure 2E**).

**Figure 3:**
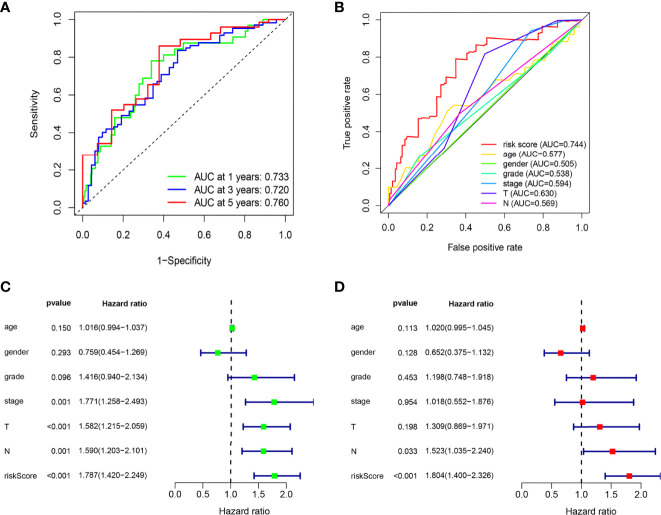
The ARG prognostic signature shows good predictive performance in the internal training dataset. **(A)** The ROC curves of the ARG model for the 1-, 3-, and 5-year survival rates, and the corresponding AUC was 0.733, 0.720, and 0.760. **(B)** The AUC value of the risk score (0.744) showed superior predictive performance than age (0.577), gender (0.505), grade (0.538), stage (0.594), T stage (0.630), and N stage (0.569). **(C)** The risk score was a significant dangerous factor to influence the OS with HR = 1.787 (95% CI = 1.420–2.249, p <0.001). Other clinical factors such as stage with HR = 1.771 (95% CI = 1.258–2.439, p = 0.001), T stage with HR = 1.582 (95% CI = 1.215–2.059, p < 0.001), and N stage with HR = 1.590 (95% CI = 1.203–2.101, p = 0.001) were significant as well. **(D)** The risk score had excellent independent prediction ability with HR = 1.804 (95% CI = 1.400–2.326, p < 0.001) in multivariate Cox regression analysis.

The ROC curves of the model showed a relatively good property, and the corresponding AUCs for 1-, 3-, and 5-year survival were 0.733, 0.720, and 0.760, which are displayed in **Figure 3A**. Univariate analysis results illustrated that the risk score (p < 0.001) significantly correlated with survival time as well as clinical stage (p = 0.001), N stage (p = 0.001), and T stage (p < 0.001) (**Figure 3C**). Then, the multivariable analysis also showed that the risk score was the best independent predictor (p < 0.001) as shown in **Figure 3D**. Moreover, the AUC of the risk score was the highest compared to other factors such as age, gender, clinical stage, and T and N stage, which demonstrated the superior forecasting performance of our gene model (**Figure 3B**).

### External Validation of the ARG Model

**Figure 4:**
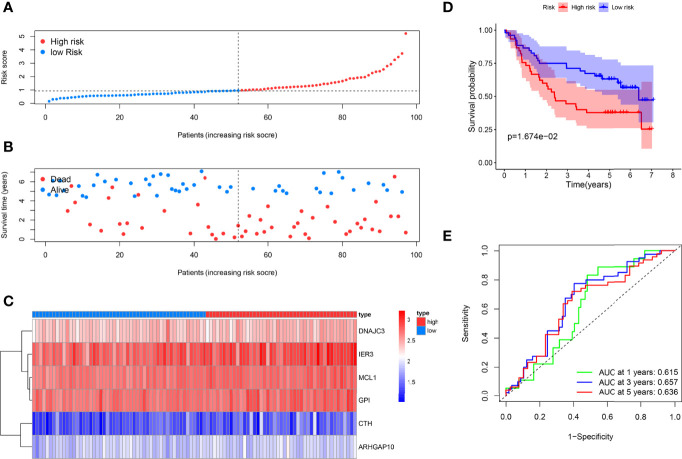
External testing of the ARG model. **(A)** Patient with different risks were separated into two groups *via* the same median risk score in the internal database. **(B)** Patients in the external dataset with different survival times and statuses were arranged by the increasing risk score from left to right. **(C)** The heatmap of the six risk ARGs in the external database showed the same expression pattern. **(D)** Kaplan–Meier analysis showed different prognoses between the two groups as well. **(E)** The ROC curves of the models for survival rate in the external testing dataset.

As supplements of **Figure 2**, we did the external validation of the ARG signature. The patients were divided into two groups in the external testing dataset with the same method as the internal database (**Figure 4A**). **Figure 4B** shows a scattergram of risk score, survival time, and status for the external testing dataset. The six ARGs presented a similar expression profile in the heatmap (**Figure 4C**). The K-M analysis presented noticeably different survival times between the two groups (**Figure 4D**). The ROC curve and corresponding AUCs in the external testing dataset were showed in (**Figure 4E**).

### Nomogram to Predict Survival Probability of OSCC

**Figure 5:**
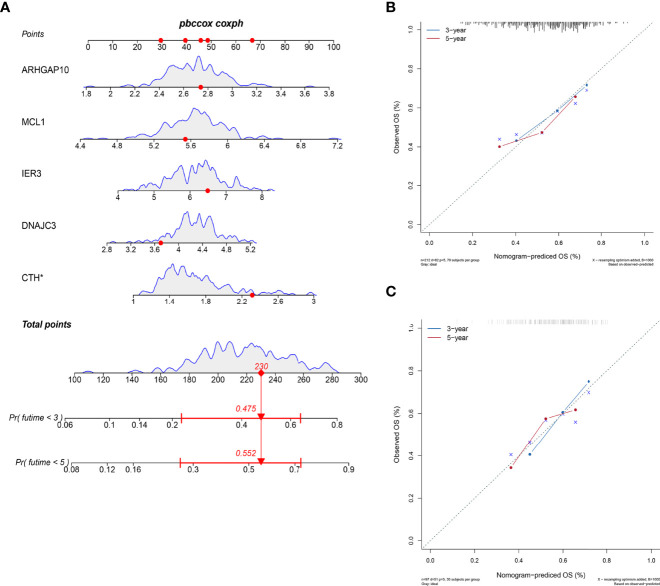
Nomogram to predict the survival probability. **(A)** The six ARGs formed the prognostic nomogram that can be used to calculate 3- and 5-year survival rates. The expression values of each gene were assigned to a unique point. The total points of six genes from one patient correspond to the specific survival rate of 3 or 5 years. **(B)** The calibration plots showed that the predicted OS of nomogram is close to the observed OS rate in the internal dataset. **(C)** The calibration plots showed the same accuracy of nomogram in the external dataset.

We developed a nomogram to estimate the survival time of OSCC quantificationally. As shown in **Figure 5A**, six ARGs were appointed with specific points according to its contribution to survival. The calibration curves to predict the survival rate were closer to diagonal, which showed an excellent effect in both the internal and external datasets (**Figures 5B, C** **)**.

### Immune Cell Infiltration Between Low-Risk and High-Risk Groups

**Figure 6:**
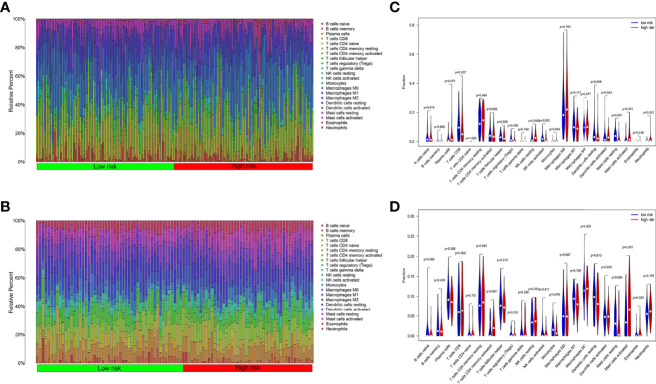
Tumor immune cell infiltration analysis. **(A)** Ratio of 22 kinds of immune cells infiltrated in OSCC tumor samples from TCGA. **(B)** Ratio of 22 kinds of immune cells infiltrated in OSCC tumor samples from GEO. **(C)** The ratio of immune cells in the high-risk group is different from the low group in TCGA. **(D)** The ratio of immune cells in the high-risk group is different from the low group in GEO.

We analyzed the component of 22 immune cells in tumor samples. Whether in the internal database or in the external database, great differences of immune cell infiltration were detected in the two groups divided by the median risk score (**Figures 6A, B** **)**. The immune cells patiently played an appropriate role in some way through the six ARGs.

**Figure 7:**
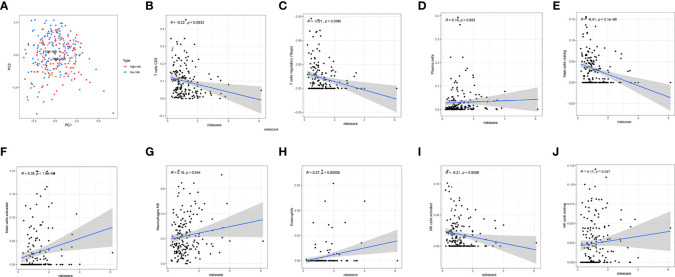
Correlation analysis of immune cell infiltration and risk scores. **(A)** PCA analysis showed a distinct immune cell infiltration pattern in two groups. **(B–J)** Correlation analysis of risk scores and nine types of the immune cells.

In TCGA internal database, the infiltration amount of NK cells resting, mast cells activated, dendritic cells activated, and eosinophils in the high-risk group were much more abundant. However, T-cells regulating (Tregs), T-cells CD8, mast cells resting, and NK cells activated in the high-risk group were much less obvious (**Figure 6C**). In the GEO external validating database, the infiltration of eosinophils and Tregs also showed the same tendency between the two groups (**Figure 6D**). Then, the correlation analysis further shed light on the relevance of risk score and nine immune cells (**Figure 7**). Plasma cells, macrophages M0, mast cells activated, eosinophils, and NK cells resting had positive correlations with the risk score. In contrast, T cells CD8, Tregs, mast cells resting, and NK cells activated correlated with the risk score in a negative way. Based on the formula of risk score, we knew that the correlation of genes and immune cells is consistent with the correlation of risk score and immune cells. Therefore, the higher the expression of risk genes, the higher the ratios of plasma cells, macrophages M0, mast cells activated, eosinophils, and NK cells infiltrated in the tumor.

### Functional Enrichment Analysis and Correlation Network of the Six Prognostic ARGs

**Figure 8:**
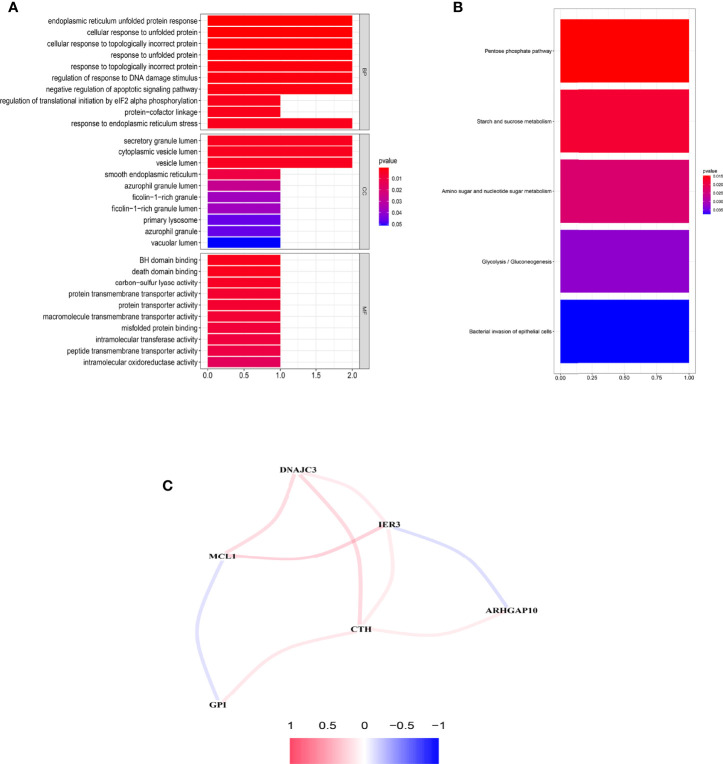
Functional enrichment analysis and the construction of network. **(A)** GO analysis of the six prognostic ARGs. **(B)** KEGG analysis of the six prognostic ARGs. **(C)** Correlation network of the six prognostic ARGs. Red means a positive interaction, and blue indicates a negative interaction.

Significant enrichments were found in cellular response to unfolded proteins, endoplasmic reticulum unfolded protein response, cellular response to topologically incorrect proteins, response to unfolded proteins, and so on (*p* < 0.05, FDR < 0.05) (**Figure 8A**). Moreover, KEGG analysis demonstrated that these ARGs were prominently enriched in nucleotide and sugar metabolism, response to unfolded proteins, glycolysis/gluconeogenesis, starch and sucrose metabolism, and bacterial invasion of epithelial cells (**Figure 8B**).

An interaction network of the potential correlation between the six ARGs was established. The network contained nine edges and six nodes (CTH, DNAJC3, IER3, MCL1, ARHGAP10, GPI), as shown in **Figure 8C**. The correlation between MCL1, GPI, IER3, and ARHGAP10 was negative, while the remaining correlations were positive.

### Unsupervised Clustering Analysis

**Figure 9:**
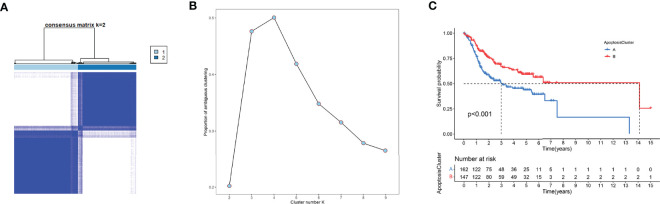
Unsupervised clustering analysis. **(A)** The clustering heatmap relevant to the consensus matrix for k = 2 acquired by consensus clustering. **(B)** Corresponding changes in area under proportion of ambiguous cluster curve with k from 2 to 9. **(C)** K-M analysis demonstrated an obvious survival difference in two apoptosis clusters.

Unsupervised clustering analysis was used to divide patient subgroups with different apoptosis modification patterns based on the expression of six ARGs. The clustering heatmap and changes in area under the proportion of the ambiguous cluster curve are shown in **Figures 9A** **, B**. One hundred sixty-two cases in TCGA-OSCC and GSE41613 OSCC cohorts were assigned to apoptosis cluster A, while 147 cases were included in cluster B. The survival analysis showed a significant survival difference between two apoptosis clusters (log-rank p value <0.001) (**Figure 9C**).

### Pan-Cancer Analysis

In order to have a more comprehensive understanding of these six risk genes with various types of cancer, we conducted some work of pan-cancer analysis. The expression of genes in pan-cancer is shown in a heatmap of (**Supplementary Figure 1**). The expression level of genes is specific to cancer species. We also get the hazard ratio of each gene in different cancers (**Supplementary Figure 2**).

### Immunohistochemical Validation

**Figure 10:**
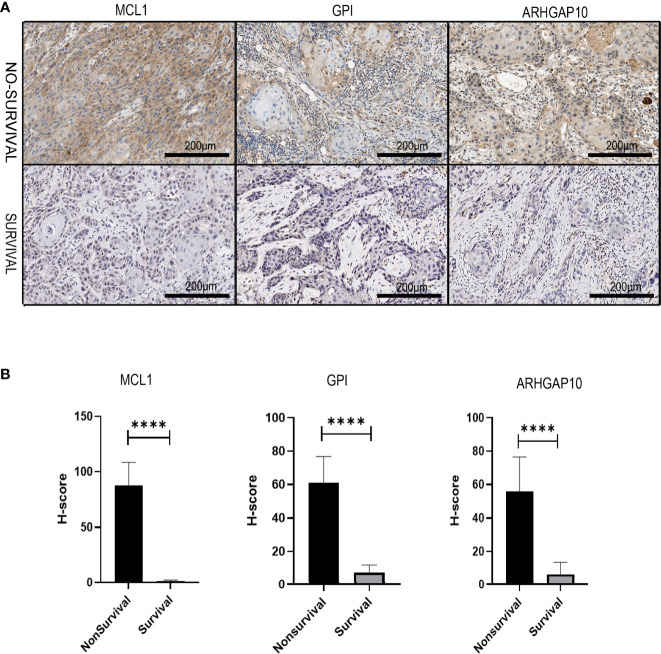
The experiment validation of the apoptosis-related prognostic gene model. **(A)** The protein expressions of three apoptosis-related prognostic genes were dramatically different in dead and alive patients who had oral cancer 3 years ago. **(B)** Corresponding H-score of protein expression of three apoptosis-related prognostic genes. **** means *p* < 0.0001.

We collected the pathological samples from the non-survival and survival patients. MCL1, GPI, and ARHGAP10 were strongly positive in the samples from non-survival patients and negative in the survival groups (**Figure 10A**). The semiquantitative comparison of the H-score between two groups also showed that these three genes were expressed more highly in non-survival patients (**Figure 10B**). Therefore, our experiment also validated that MCL1, GPI, and ARHGAP10 were risk genes which can predict the prognosis of OSCC.

## Discussion

In the present study, we acquired information and gene sets from 213 OSCC patients for analysis, and p values <0.05 were singled out for further analyses. We constructed a model made up of six biomarkers to estimate the survival time of OSCC patients. In accordance with the risk score, OSCC patients were successfully allocated into low- and high-risk groups with distinct prognosis. Moreover, the ARG model was an independent forecasting factor with better efficiency compared to previous traditional clinical factors. Moreover, we used the six genes to build a novel nomogram that can provide superior estimation of OS. However, the underlying mechanisms of these results still need to be further investigated.

In recent years, numerous studies have confirmed the relationship between apoptosis and cancer (1, 7, 11). Apoptotic signal pathways can be changed at transcriptional, translational, and posttranslational levels in cancer cells. Several proteins, such as the BCL-2 family, have been shown to function in the process of apoptosis and cancer (18, 19). Myeloid cell leukemia-1 (MCL1), which is well known for its anti-apoptotic role in the Bcl-2 family, is a distinct cell regulatory protein. MCL1 is required for cell survival, differentiation, and maintenance (20). Recent studies have also revealed that MCL1 is a therapeutic prospective target in cancers (21, 22). Knocking down MCL1 could induce the apoptosis of OSCC cells through increasing the sensitivity to drugs. Moreover, MCL1 antagonist Sabutoclax could increase cancer cell death in OSCC as well (23).

Glucose-6-phosphate isomerase (GPI) is one of the members in the glucose phosphate isomerase protein family. GPI plays a crucial part in the process of glycolysis and gluconeogenesis (24, 25). Earlier studies showed that GPI could be used as a prognostic biomarker in cancer because of its important work in the cell cycle (26, 27). In our study, GPI was a risk gene because a higher expression was associated with poor prognosis. However, the role of GPI in OSCC needs to be explored in detail.

ARHGAP10 is a member of the RhoGAP protein group, which can convert the active form to inactive form (28). Downregulating the expression of ARHGAP10 could lead to a more advanced stage and a higher Ki-67 index in breast cancer (29). Luo pointed out that ARHGAP10 may serve as a tumor inhibitor through suppressing adhesion, migration, and invasion of the ovarian cancer cells (30). Teng also found the same phenomenon in lung cancer (31). These results seem opposite to our analysis and IHC validation in OSCC. However, in prostate cancer, Hua Gong claimed that a high expression of ARHGAP10 correlates with poor prognosis, which supports our result (32). The mechanisms underlying the paradox that the same molecule plays a different role in different cancers need to be further studied.

The immune microenvironment has great impact on the tumorigenesis of OSCC (33). TIL plays a key part in cancer genesis, especially in immune evasion (34, 35). In our research, the correlation analysis sheds light on the connection of risk score and immune cells. It is meaningful to explore the mutual effect of tumor cells and immune cells. Studies about CAR T cells targeting solid tumors (36), B cell-based immunotherapy for lung cancer (37), dendritic cell-based vaccination, and so on, provided a new insight to develop more effective therapy regimens (38). Other research focused on the TILs and OSCC also revealed similar results (39), although how the TILs interact with these six prognostic ARGs specifically still requires deeper research.

Some limitations still need to be considered in our study. The construction and validation of prognostic signature were based on the previous published database. If we could use the data from a real-life clinical cohort, maybe we can acquire more reliable and meaningful discoveries. The six prognostic ARGs and nomogram should be tested in subsequent studies and clinical trials.

In conclusion, we identified six risk biomarkers, namely, MCL1, GPI, ARHGAP10, CTH, DNAJC3, and IER3, associated with prognosis of OSCC through a comprehensive bioinformatics analysis based on TCGA database and apoptosis-related gene sets. The gene signature and nomogram might provide a precise prognostic prediction of OSCC, which will help clinicians to formulate individual treatment strategies for their patients. These genes identified could also be therapeutic targets for OSCC.

## Data Availability Statement

Publicly available datasets were analyzed in this study. These data can be found here: OSCC cohort from TCGA database, https://portal.gdc.cancer.gov/; GSE41613 from GEO database, https://www.ncbi.nlm.nih.gov/geo.

## Ethics Statement

The studies involving human participants were reviewed and approved by the Research Ethics Committee of Guangdong Provincial Hospital & Guangdong Academy of Medical Sciences. Written informed consent was provided by the participating patient(s).

## Author Contributions

SW, LZ, and GL conceived and contributed to the study concept and design. SZ contributed to the data acquisition. SW collected the specimens and developed the data analysis workflows. SW, ZL, and JM performed the data analysis and interpreted the results. ZL and JM finished the immunohistochemical stain. SW wrote the manuscript. SZ mended the manuscript. GL and LZ supervised the study. All authors contributed to the article and approved the submitted version.

## Funding

This research was supported by grant 2019A1515012218 (SZ) funded by the Foundation and Applied Foundation Research Fund project of Guangdong Province and grant 201904010008 funded by Guangzhou Science and Technology Project.

## Conflict of Interest

The authors declare that the research was conducted in the absence of any commercial or financial relationships that could be construed as a potential conflict of interest.

## Publisher’s Note

All claims expressed in this article are solely those of the authors and do not necessarily represent those of their affiliated organizations, or those of the publisher, the editors and the reviewers. Any product that may be evaluated in this article, or claim that may be made by its manufacturer, is not guaranteed or endorsed by the publisher.

## Supplementary Material

Supplementary Figure 1 The heatmap showed expression profile of the 6 risk genes in pan-cancer.

Supplementary Figure 2 Forest plot showed the correlation of 6 risk genes with the prognosis in pan-cancer.

## References

[1] Sung H Ferlay J Siegel RL Laversanne M Soerjomataram I Jemal A. Global Cancer Statistics 2020: GLOBOCAN Estimates of Incidence and Mortality Worldwide for 36 Cancers in 185 Countries. CA Cancer J Clin (2021) 71(3):209–49. doi: 10.3322/caac.21660 33538338

[2] D’souza S Addepalli V. Preventive Measures in Oral Cancer: An Overview. BioMed Pharmacother (2018) 107(May):72–80. doi: 10.1016/j.biopha.2018.07.114 30081204

[3] Weber M Wehrhan F Baran C Agaimy A Büttner-Herold M Kesting M. Prognostic Significance of PD-L2 Expression in Patients With Oral Squamous Cell Carcinoma-A Comparison to the PD-L1 Expression Profile. Cancer Med (2019) 8(3):1124–34. doi: 10.1002/cam4.1929 PMC6434218 30659749

[4] Bettendorf O Piffkò J Bànkfalvi A. Prognostic and Predictive Factors in Oral Squamous Cell Cancer: Important Tools for Planning Individual Therapy? Oral Oncol (2004) 40(2):110–9. doi: 10.1016/j.oraloncology.2003.08.010. PMID:14693233 14693233

[5] Elmore S. Apoptosis: A Review of Programmed Cell Death. Toxicol Pathol (2007) 35(4):495–516. doi: 10.1080/01926230701320337 17562483 PMC2117903

[6] Liu J Liu W Yang H. Balancing Apoptosis and Autophagy for Parkinson's Disease Therapy: Targeting BCL-2. ACS Chem Neurosci (2019) 10(2):792–802. doi: 10.1021/acschemneuro.8b00356 30400738

[7] Mohammad RM Muqbil I Lowe L Yedjou C Hsu HY Lin LT. Broad Targeting of Resistance to Apoptosis in Cancer. Semin Cancer Biol (2015) 35 Suppl(0):S78–S103. doi: 10.1016/j.semcancer.2015.03.001 25936818 PMC4720504

[8] Strasser A Vaux DL. Cell Death in the Origin and Treatment of Cancer. Mol Cell (2020) 78(6):1045–54. doi: 10.1016/j.molcel.2020.05.014 32516599

[9] Singh R Letai A Sarosiek K. Regulation of Apoptosis in Health and Disease: The Balancing Act of BCL-2 Family Proteins. Nat Rev Mol Cell Biol (2019) 20(3):175–93. doi: 10.1038/s41580-018-0089-8 PMC7325303 30655609

[10] Hu J Cao J Topatana W Juengpanich S Li S Zhang B. Targeting Mutant P53 for Cancer Therapy: Direct and Indirect Strategies. J Hematol Oncol (2021) 14(1):157. doi: 10.1186/s13045-021-01169-0 34583722 PMC8480024

[11] Goldar S Khaniani MS Derakhshan SM Baradaran B. Molecular Mechanisms of Apoptosis and Roles in Cancer Development and Treatment. Asian Pac J Cancer Prev (2015) 16(6):2129–44. doi: 10.7314/apjcp.2015.16.6.2129 25824729

[12] Boice A Bouchier-Hayes L. Targeting Apoptotic Caspases in Cancer. Biochim Biophys Acta Mol Cell Res (2020) 1867(6):118688. doi: 10.1016/j.bbamcr.2020.118688 32087180 PMC7155770

[13] Sasahira T Kirita T. Hallmarks of Cancer-Related Newly Prognostic Factors of Oral Squamous Cell Carcinoma. Int J Mol Sci (2018) 19(8):2413. doi: 10.3390/ijms19082413 PMC6121568 30115834

[14] Li J Ma S. Time-Dependent ROC Analysis Under Diverse Censoring Patterns. Stat Med (2011) 30(11):1266–77. doi: 10.1002/sim.4178 21538452

[15] Newman AM Liu CL Green MR Gentles AJ Feng W Xu Y. Robust Enumeration of Cell Subsets From Tissue Expression Profiles. Nat Methods (2015) 12(5):453–7. doi: 10.1038/nmeth.3337 PMC4739640 25822800

[16] Wu T Hu E Xu S Chen M Guo P Dai Z. Cluster Profiler 4.0: A Universal Enrichment Tool for Interpreting Omics Data. Innovation (N Y). (2021) 2(3):100141. doi: 10.1016/j.xinn.2021.100141 PMC8454663 34557778

[17] Ng KL Ellis RJ Samaratunga H Morais C Gobe GC Wood ST. Utility of Cytokeratin 7, S100A1 and Caveolin-1 as Immunohistochemical Biomarkers to Differentiate Chromophobe Renal Cell Carcinoma From Renal Oncocytoma. Transl Androl Urol (2019) 8(Suppl 2):S123–37. doi: 10.21037/tau.2018.11.02 PMC6559932 31236330

[18] Pistritto G Trisciuoglio D Ceci C Garufi A D'Orazi G. Apoptosis as Anticancer Mechanism: Function and Dysfunction of its Modulators and Targeted Therapeutic Strategies. Aging (Albany NY) (2016) 8(4):603–19. doi: 10.18632/aging.100934 PMC4925817 27019364

[19] Knight T Luedtke D Edwards H Taub JW Ge Y. A Delicate Balance - The BCL-2 Family and Its Role in Apoptosis, Oncogenesis, and Cancer Therapeutics. Biochem Pharmacol (2019) 162:250–61. doi: 10.1016/j.bcp.2019.01.015 30668936

[20] Sancho M Leiva D Lucendo E Orzáez M. Understanding MCL1: From Cellular Function and Regulation to Pharmacological Inhibition. FEBS J (2021). doi: 10.1111/febs.16136. Epub ahead of print PMC9787394 34310025

[21] Wu X Luo Q Liu Z. Ubiquitination and Deubiquitination of MCL1 in Cancer: Deciphering Chemoresistance Mechanisms and Providing Potential Therapeutic Options. Cell Death Dis (2020) 11(7):556. doi: 10.1038/s41419-020-02760-y 32699213 PMC7376237

[22] Wei AH Roberts AW Spencer A Rosenberg AS Siegel D Walter RB. Targeting MCL-1 in Hematologic Malignancies: Rationale and Progress. Blood Rev (2020) 44:100672. doi: 10.1016/j.blre.2020.100672 32204955 PMC7442684

[23] Maji S Samal SK Pattanaik L Panda S Quinn BA Das SK. Mcl-1 Is an Important Therapeutic Target for Oral Squamous Cell Carcinomas. Oncotarget (2015) 6(18):16623–37. doi: 10.18632/oncotarget.3932 PMC4599294 26009874

[24] Aguilera-Romero A Sabido-Bozo S Lopez S Cortes-Gomez A Rodriguez-Gallardo S Perez-Linero AM. Determination of the Lipid Composition of the GPI Anchor. PloS One (2021) 16(8):e0256184. doi: 10.1371/journal.pone.0256184 34388214 PMC8362999

[25] Lebreton S Zurzolo C Paladino S. Organization of GPI-Anchored Proteins at the Cell Surface and Its Physiopathological Relevance. Crit Rev Biochem Mol Biol (2018) 53(4):403–19. doi: 10.1080/10409238.2018.1485627 30040489

[26] Han J Deng X Sun R Luo M Liang M Gu B. GPI Is a Prognostic Biomarker and Correlates With Immune Infiltrates in Lung Adenocarcinoma. Front Oncol (2021) 11:752642. doi: 10.3389/fonc.2021.752642 34912709 PMC8666546

[27] Gamage DG Hendrickson TL. GPI transamidase And GPI Anchored Proteins: Oncogenes and Biomarkers for Cancer. Crit Rev Biochem Mol Biol (2013) 48(5):446–64. doi: 10.3109/10409238.2013.831024 23978072

[28] Sekiguchi M Sobue A Kushima I Wang C Arioka Y Kato H. ARHGAP10, Which Encodes Rho GTPase-Activating Protein 10, Is a Novel Gene for Schizophrenia Risk. Transl Psychiatry (2020) 10(1):247. doi: 10.1038/s41398-020-00917-z 32699248 PMC7376022

[29] Li Y Zeng B Li Y Zhang C Ren G. Downregulated Expression of ARHGAP10 Correlates With Advanced Stage and High Ki-67 Index in Breast Cancer. PeerJ (2019) 7:e7431. doi: 10.7717/peerj.7431 31396458 PMC6679923

[30] Luo N Guo J Chen L Yang W Qu X Cheng Z. ARHGAP10, Downregulated in Ovarian Cancer, Suppresses Tumorigenicity of Ovarian Cancer Cells. Cell Death Dis (2016) 7(3):e2157. doi: 10.1038/cddis.2015.401 27010858 PMC4823924

[31] Teng JP Yang ZY Zhu YM Ni D Zhu ZJ Li XQ. The Roles of ARHGAP10 in the Proliferation, Migration and Invasion of Lung Cancer Cells. Oncol Lett (2017) 14(4):4613–8. doi: 10.3892/ol.2017.6729 PMC5592856 28943961

[32] Gong H Chen X Jin Y Lu J Cai Y Wei O. Expression of ARHGAP10 Correlates With Prognosis of Prostate Cancer. Int J Clin Exp Pathol (2019) 12(10):3839–46. PMC6949770 31933772

[33] Lei X Lei Y Li JK Du WX Li RG Yang J. Immune Cells Within the Tumor Microenvironment: Biological Functions and Roles in Cancer Immunotherapy. Cancer Lett (2020) 470:126–33. doi: 10.1016/j.canlet.2019.11.009 31730903

[34] Jie HB Srivastava RM Argiris A Bauman JE Kane LP Ferris RL. Increased PD-1+ and TIM-3+ TILs During Cetuximab Therapy Inversely Correlate With Response in Head and Neck Cancer Patients. Cancer Immunol Res (2017) 5(5):408–16. doi: 10.1158/2326-6066.CIR-16-0333 PMC5497750 28408386

[35] Chen L Diao L Yang Y Yi X Rodriguez BL Li Y. CD38-Mediated Immunosuppression as a Mechanism of Tumor Cell Escape From PD-1/PD-L1 Blockade. Cancer Discovery (2018) 8(9):1156–75. doi: 10.1158/2159-8290.CD-17-1033 PMC6205194 30012853

[36] Martinez M Moon EK. CAR T Cells for Solid Tumors: New Strategies for Finding, Infiltrating, and Surviving in the Tumor Microenvironment. Front Immunol (2019) 10:128. doi: 10.3389/fimmu.2019.00128 30804938 PMC6370640

[37] Wang SS Liu W Ly D Xu H Qu L Zhang L. Tumor-Infiltrating B Cells: Their Role and Application in Anti-Tumor Immunity in Lung Cancer. Cell Mol Immunol (2019) 16(1):6–18. doi: 10.1038/s41423-018-0027-x 29628498 PMC6318290

[38] Sabado RL Balan S Bhardwaj N. Dendritic Cell-Based Immunotherapy. Cell Res (2017) 27(1):74–95. doi: 10.1038/cr.2016.157 28025976 PMC5223236

[39] Succaria F Kvistborg P Stein JE Engle EL McMiller TL Rooper LM. Characterization of the Tumor Immune Microenvironment in Human Papillomavirus-Positive and -Negative Head and Neck Squamous Cell Carcinomas. Cancer Immunol Immunother (2021) 70(5):1227–37. doi: 10.1007/s00262-020-02747-w PMC8188514 33125511

