# Supplementary figures and images for: Identification and Validation of an Apoptosis-Related Gene Prognostic Signature for Oral Squamous Cell Carcinoma
Source: Front Oncol. 2022 Jun 13;12:889049. doi: 10.3389/fonc.2022.889049 (PMC9235536; doi:10.3389/fonc.2022.889049)

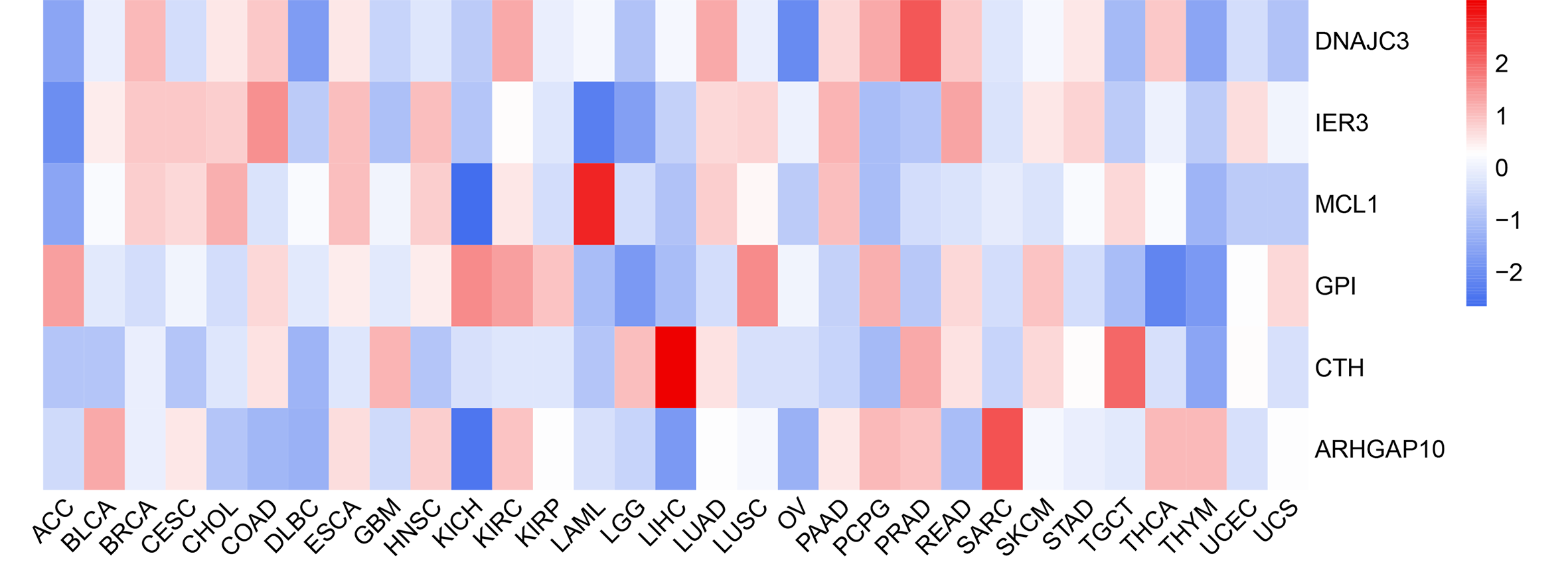

Supplement: Supplementary Figure 1 — The heatmap showed expression profile of the 6 risk genes in pan-cancer. [file Image_1.tif]

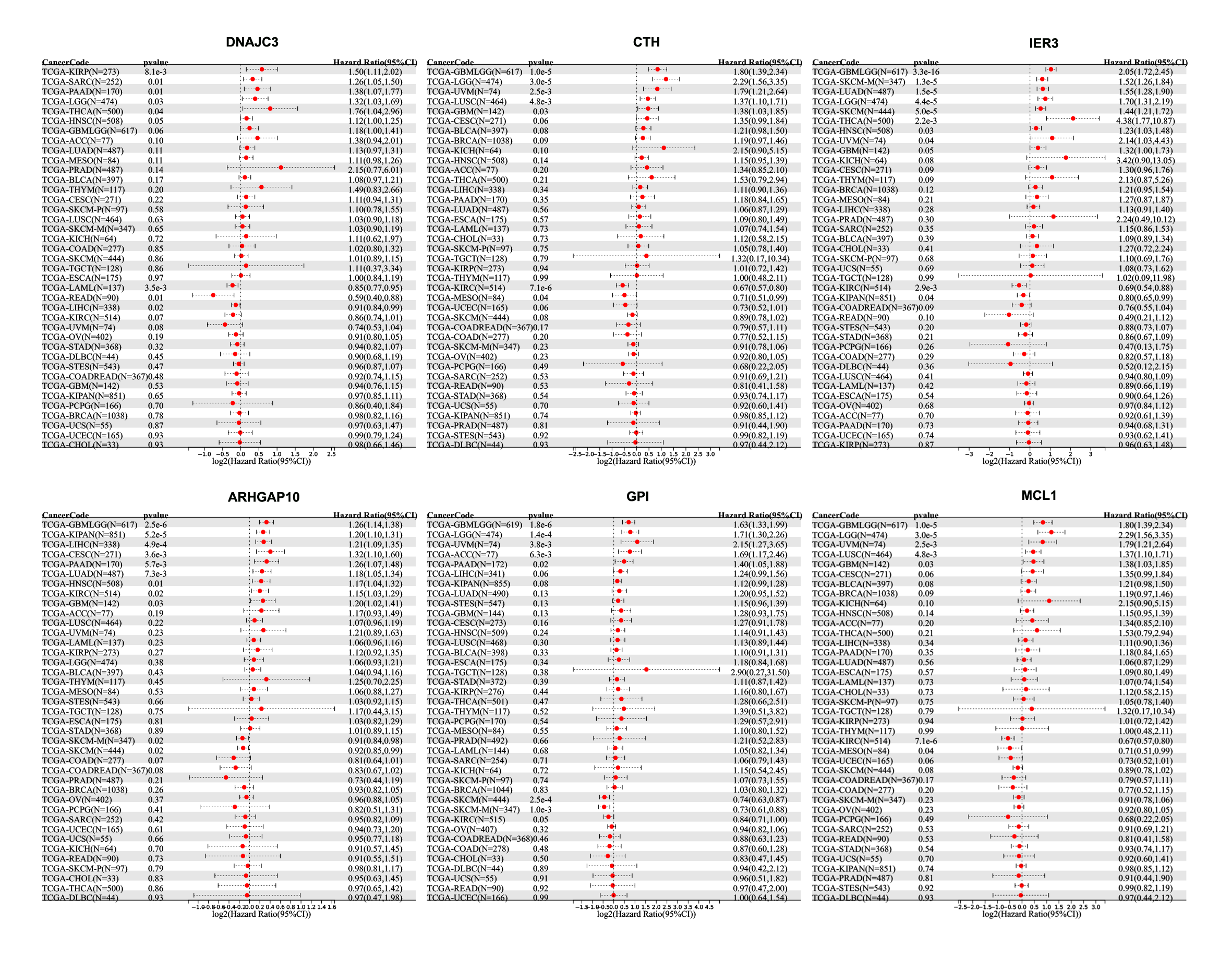

Supplement: Supplementary Figure 2 — Forest plot showed the correlation of 6 risk genes with the prognosis in pan-cancer. [file Image_2.tif]
